# Mechanism and Selectivity of Copper-Catalyzed Bromination of Distal C(sp^3^)–H Bonds

**DOI:** 10.1021/acs.organomet.2c00554

**Published:** 2023-02-23

**Authors:** Manjaly J. Ajitha, Brandon E. Haines, Djamaladdin G. Musaev

**Affiliations:** Cherry L. Emerson Center for Scientific Computation, Emory University, 1515 Dickey Drive, Atlanta, Georgia 30322, United States

## Abstract

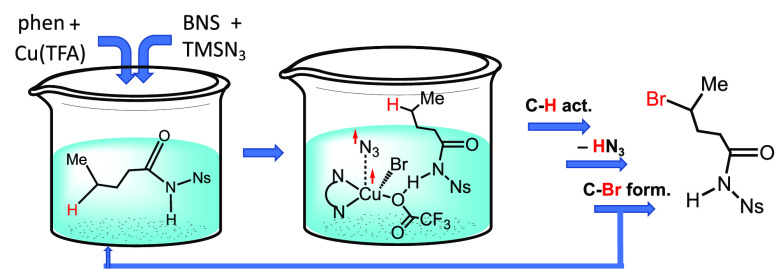

Unactivated C(sp^3^)–H bonds are the most challenging substrate class for transition metal-catalyzed C–H halogenation. Recently, the Yu group [Liu, T.; Myers, M. C.; Yu, J. Q. *Angew. Chem., Int. Ed.***2017**, *56* (1), 306–309] has demonstrated that a Cu^II^/phenanthroline catalyst and BrN_3_, generated *in situ* from NBS and TMSN_3_ precursors, can achieve selective C–H bromination distal to a directing group. The current understanding of the mechanism of this reaction has left numerous questions unanswered. Here, we investigated the mechanism of Cu-catalyzed C(sp^3^)–H bromination with distal site selectivity using density functional theory calculations. We found that this reaction starts with the Br-atom transfer from BrN_3_ to the Cu center that occurs via a small energy barrier at the singlet–triplet state seam of crossing. In the course of this reaction, the presence of the N–H bond in the substrate is critically important and acts as a directing group for enhancing the stability of the catalyst–substrate interaction and for the recruitment of the substrate to the catalyst. The required C-centered radical substrate formation occurs via direct C–H dehydrogenation by the Cu-coordinated N_3_ radical, rather than via the previously proposed N–H bond dehydrogenation and then the 1,5-H transfer from the γ-(C–H) bond to the N-radical center pathway. The C–H bond activation by the azide radical is a regioselectivity-controlling step. The following bromination of the C-centered radical by the Cu-coordinated bromine completes the product formation. This reaction step is the rate-limiting step, occurs at the singlet-to-triplet state seam of the crossing point, and is exergonic.

## 1 Introduction

Selective functionalization of “inert” C–H bonds of broadly accessible hydrocarbons and heterocycles in an environmentally benign, less expensive, and atom-economical fashion has revolutionized the synthesis of desirable pharmaceuticals, agrochemicals, materials, and fuels.^1−16^ Despite the latest advances in this field, the development of selective and highly effective C–H halogenation remains a challenge.^17−43^ Alkyl halides are important synthons for the total synthesis of pharmaceutically relevant compounds and are vulnerable to a variety of functional group transformations.^44−46^ Traditionally, they are synthesized via classical functional group transformation with the corresponding alcohols, olefins, and carboxylic acids as precursors or by radical reactions with poor selectivity.^47,48^

An emergent approach for accessing these synthetically valuable compounds is transition metal (TM)-catalyzed C–H functionalization, i.e., C–H halogenation, whereby a TM complex, ligand, halogen source, and/or oxidant are used to directly transform a C–H bond into a C–X bond, where X = F, Cl, Br, or I.^48^ This approach is attractive because it holds the potential to minimize the number of steps in a synthetic route and enable late-stage functionalization of complex, precious molecules. Currently, broadly utilized synthetic strategies enabling the transformation of “inert” C–H bonds into C–halogen bonds are the use of TM catalysts in connection with the F–N reagents [such as *N*-fluorobenzenesulfonimide (NFSI), *N*-fluoropyridinium salts (NFPy), and 1-chloromethyl-4-fluoro-1,4-diazoniabicyclo[2.2.2]octane bis(tetrafluoroborate) (Selectfluor)]^49−78^ and the photochemical halogenation of amines by F–N, Cl–N, and Br–N precursors.^79^ Ongoing research has characterized these versatile F–N reagents as both halogen sources and catalysts.^53−55^ Furthermore, it has been shown that the utilized transition metal complexes can act as both a promoter and/or a catalyst. For example, Lectka and co-workers^58^ have shown that in the earth-abundant Cu(I)-mediated C–H bond fluorination of aliphatic substrates by Selectfluor, the Cu complex promotes the formation of the dicationic amynyl radical catalyst. In contrast, Sarpong, Musaev, and co-workers^63−65^ have demonstrated that the Cu(I)- and Ag(I)-mediated C–C deconstructive C–H fluorination in N-benzoylated cyclic amines by Selectfluor is a two-state reactivity (TSR) event that proceeds via the F-atom coupled electron transfer (FCET) pathway and leads to the oxidative addition coupled electron transfer (OA+ET) product. In spite of these and other advances, the search for more general C–H halogenation strategies enabling (a) identification of alternative catalysts and halogenation reagents that are less expensive, (b) improvement of the functional group compatibility of the existing processes, and (c) access to a broader scope of substrates is still an active research direction with greater fundamental and practical potential.

**Scheme 1:**
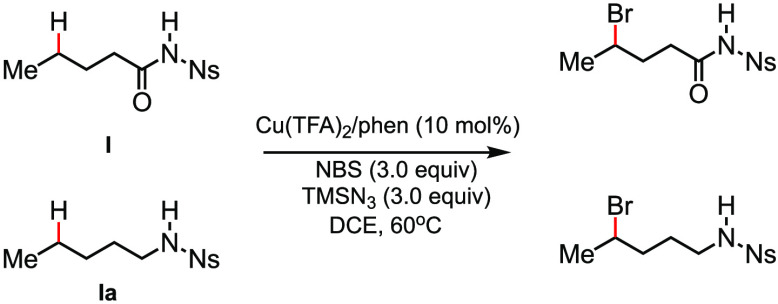
Cu/Phenanthroline-Catalyzed Bromination of γ-C–H Bonds of Aliphatic Amides and δ-C–H Bonds of Alkyl Amines

Unactivated C(sp^3^)–H bonds are arguably the most challenging substrate class for TM-catalyzed C–H halogenation due to their ubiquity and general chemical inertness.^80−85^ However, motivated by the upside of this approach, the Yu group has demonstrated that a Cu^II^/phenanthroline catalyst and BrN_3_, which is generated *in situ* from NBS and TMSN_3_ precursors, can achieve selective C–H bromination distal to a directing group (see Scheme 1). This reaction affords γ-selectivity of aliphatic amides and δ-selectivity of alkyl amines.^86^

**Scheme 2:**
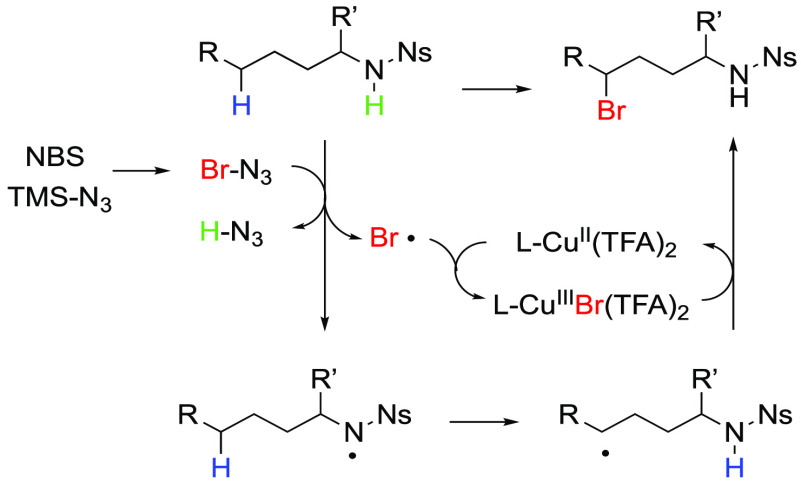
Mechanism for Cu-Catalyzed Distal C–H Bromination with BrN_3_ Proposed from the Available Experimental Data (reproduced with permission from ref (86), copyright 2017 John Wiley and Sons)

On the basis of the available experimental data, the proposed mechanism (see Scheme 2) for this reaction includes the following steps: (i) formation of BrN_3_*in situ* from NBS and TMSN_3_ under the reaction conditions, (ii) homolytic cleavage of the Br–N_3_ bond to form an azide radical that performs abstraction of a hydrogen atom from the N–H bond of the substrate with concurrent oxidation of the Cu^II^ catalyst by a bromine radical, (iii) 1,5- or 1,6-H-atom abstraction by the N-centered radical to form a C-centered radical intermediate, and (iv) abstraction of the bromine by the C-centered radical, which reduces the Cu^III^ intermediate to regenerate the Cu^II^ catalyst and leads to the brominated product.^86^ The absence of cyclized product and radical clock experiments indicates that oxidation of the C-centered radical to a cation is slow under these conditions.^86^

However, this current understanding of the mechanism must be improved to further capitalize on the unique reactivity and selectivity of this reaction. The identity of the active catalyst is unknown because both Cu^I^ and Cu^II^ catalyst precursors give the product.^86^ In addition, the mechanism for determining the site selectivity of C–H bromination is unclear. Lastly, the use of BrN_3_ is not ideal because it is explosive and toxic.^87^ A better understanding of its role in the reaction will aid in the design of a safer and more efficient halogenating reagent. Therefore, further mechanistic studies will provide critical information for the development of novel catalytic C–H halogenation and C–H functionalization reactions that address the foremost challenges in the field: (i) using earth-abundant TM catalysts and (ii) achieving distal site selectivity.

With this motivation, we investigated the detailed mechanism of the Cu-catalyzed C(sp^3^)–H bromination with distal site selectivity using density functional theory (DFT) calculations to provide insight into the following: (1) the catalytically active form of the Cu catalyst, (2) the role of BrN_3_ and N_3_ radical, and (3) key mechanistic details of the reaction. We are expecting to acquire fundamental knowledge that will enlighten the development of the next generation of catalytic systems to build upon the achievements of remote C(sp^3^)–H bromination of aliphatic amines and amides toward more robust, cost-effective, safe, and selective C–H halogenation and C–H functionalization reactions.

## 2 Computational Details

The preferred mechanistic pathway has been identified by locating the intermediates and transition states at the B3LYP/Gen1 level of theory,^88−90^ where Gen1 is the Los Alamos National Laboratory double ζ (lanl2DZ) basis set^91,92^ for Cu and 6-31G(d,p) for all other atoms, along with Grimme’s empirical dispersion correction (B3LYP-D3)^93,94^ using the Gaussian 09 quantum chemistry package.^95^ Extensive conformational search for intermediates and transition states have been conducted at the same level of theory (by manual rotation of key bonds and full optimization of all degrees of freedom), but only the most stable geometries are discussed. Energies were further refined at the B3LYP-D3/Gen2 level, where Gen2 is the SDD basis set^96^ for Cu and the 6-311+G(2d,p) basis set for all other atoms. The utilized computational approach previously was shown^25,40^ to be reliable for the study of the organic and organometallic reactions similar to that investigated in this paper. The radical species were treated with the spin-unrestricted formalism, and the stability of their wave functions was confirmed. Bulk solvent effects have been incorporated for all calculations (geometry optimization, frequency calculation, and single-point energy calculations) using the self-consistent reaction field polarizable continuum model (IEF-PCM) with 1,2-dichloroethane as the solvent.^97,98^ Thermodynamic parameters have been corrected to a standard state of 1 M and 298.15 K. All transition states were confirmed by single imaginary frequencies pertaining to the reaction coordinates and were further confirmed by IRC calculations.^99^ Energies are presented as Δ*H*/Δ*G* in kilocalories per mole, while the discussion is presented on the basis of the relative Gibbs energies with respect to pre-reaction complexes, unless otherwise specified. It should also be noted that the dissociation and association of two radicals are often associated with spin crossover when the spin state of the reactants is singlet and the product is triplet or vice versa. The energy barriers for the spin-crossover process of dissociation and association of radicals are represented by the minimum energy crossing point (MECP).^100^ Here, MECPs between singlet and triplet states were located using MECPro version 1.0.3 developed by Ess and co-workers.^101^ Cartesian coordinates of all reported structures and their energies are given in the Supporting Information.

## 3 Results and Discussion

Several reaction pathways were examined computationally for this reaction.^102^ The control experiments by Yu and co-workers^86^ showed that (a) both Cu(I) and Cu(II) complexes catalyze this reaction while the use of Cu^II^(TFA)_2_ as a precatalyst provided a slightly higher reaction yield, (b) both the copper complex and phenanthroline (phen) ligand are essential for the desired bromination reaction to proceed, and (c) both γ-(C–H) bond bromination of *N*-Ns-pentanamide **I** and δ-(C–H) bond bromination of *N*-Ns-pentanamine **Ia** proceed, basically, via the same mechanism. In this work, we used both Cu^I^(TFA) and Cu^II^(TFA)_2_ as catalysts in the γ-(C–H) bond bromination of **I** and, consistent with previous experiments, found that both reactions proceed via conceptually the same mechanistic scenarios. For the sake of simplicity, below we discuss in detail only the (phen)Cu^I^(TFA) (**II**)-catalyzed γ-(C–H) bond bromination in **I**, while all calculated data for the (phen)Cu^II^(TFA)_2_-catalyzed reaction are included in the Supporting Information. Because experiments have identified BrN_3_, generated by the reaction of NBS and TMSN_3_, as an oxidant, it is conceivable to hypothesize that the (phen)Cu^I^(TFA) (**II**)-catalyzed γ-(C–H) bond bromination of *N*-Ns-pentanamide **I** is going to be initiated by the reaction of **II** with either oxidant BrN_3_ or substrate **I**, or simultaneously.^103^

Generation of the expected brominating agent, BrN_3_, from NBS and TMSN_3_ is computed to be slightly endergonic [NBS + TMSN_3_ → BrN_3_ + *N*-trimethylsilylsuccinimide (Δ*G* = 4.6 kcal/mol)]. This is the reason why several equivalents of NBS and TMSN_3_ are required under the reaction conditions.^86^ Even so, the concentration of BrN_3_ in the reaction mixture will be low. Forming BrN_3_ in this way is advantageous because it is highly reactive and explosive.^104^

### 3.1 Coordination of the Substrate versus BrN_3_ to the Cu^I^ Complex

**Figure 1:**
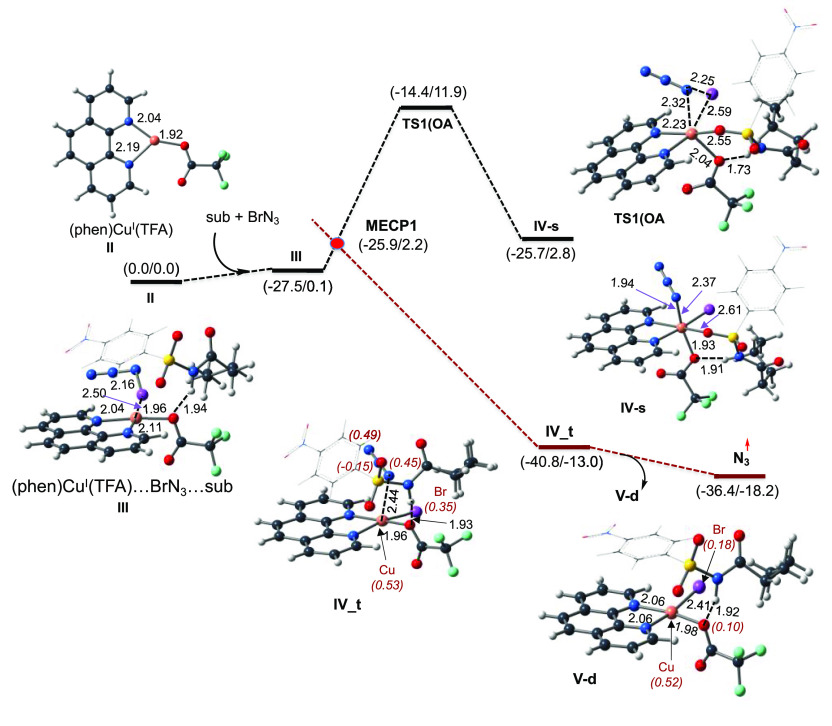
Potential energy surface (PES) of the reaction **II** + **I** + BrN_3_ leading to the **IV-s**, **IV-t**, and **V-d** complexes. The calculated Δ*H*/Δ*G* values (in parentheses) are in kilocalories per mole, and bond distances are in angstroms. The italic numbers in parentheses are the corresponding Mulliken atomic spin densities of selected atoms. Color code: black, C; blue, N; white, H; purple, Br; green, F; red, O; and orange, Cu.

We next examined the association of BrN_3_ and **I** with the (phen)Cu^I^(TFA) catalyst **II**. Coordination of **I** to **II** is exergonic by 4.5 kcal/mol. A hydrogen-bonding interaction between the amide and TFA ligand (N–H–O, 1.74 Å) is a major stabilizing factor and helps in the recruitment of the substrate to the catalyst. Coordination of BrN_3_ to **II** is endergonic by 1.2 kcal/mol. Simultaneous coordination of **I** and BrN_3_ to **II** to form the **I**–(phen)Cu^I^(TFA)–[BrN_3_] complex (**III**) (see Figure 1) is almost thermoneutral, so we continued our study from this point. In this complex, oxidant BrN_3_ is coordinated to the Cu center via its Br atom with a Cu–BrN_3_ bond distance of 2.50 Å. Interestingly, in **III**, the substrate is coordinated to the μ^2^-O center of the TFA ligand (rather than the Cu center) via the H atom of its amine group: the calculated (μ^2^-O)–HN bond distance is 1.94 Å.

We hypothesized that cleavage of the Br–N_3_ bond in **III** could occur through a two-electron oxidative addition pathway (i.e., Cu^I^ + Br–N_3_ → BrCu^III^N_3_) or a one-electron halogen transfer pathway (i.e., Cu^I^ + Br–N_3_ → Cu^II^Br + N_3_^•^). Therefore, we propose the reaction of **II** with BrN_3_, in the presence of substrate **I**, to be the first step of the reaction. Consistently, we also were able to identify two possible products of this reaction, namely, singlet state complex **IV-s** and triplet state complex **IV-t** (see Figure 1). Close examinations show that complex **IV-s** is a product of the Br–N_3_ oxidative addition to the Cu^I^ center, which requires a 11.9 kcal/mol free energy barrier at singlet state transition state **TS1(OA)**. Relative to complex **III**, in this transition state structure, the activated N_3_–Br bond is elongated to 2.25 Å (from 2.16 Å) while the both (μ^2^-O)–HN and Cu–O^3^ (from the Ns group) bonds are shortened to 1.73 and 2.55 Å, respectively. As shown in Figure 1, the overall reaction **II** + **I** + BrN_3_ → **IV-s** is endergonic by 2.8 kcal/mol. The calculated energetics of this reaction are consistent with the established concept that Cu^I^-to-Cu^III^ oxidation is an energetically uphill process.

Triplet state complex **IV-t** lies lower in free energy than reactants by 13.0 kcal/mol and, as one could expect, is a product of the abstraction of the Br atom from BrN_3_ by the Cu center of complex **III**. Indeed, close analyses of the calculated Mulliken atomic spin densities show that the Cu–Br and N_3_ units in **IV-t** possess by 0.88 and 0.79 |e| unpaired α-spins, respectively. Thus, (a) **IV-t** is a Cu^II^ intermediate, (**I**)–(phen)[Cu^II^Br]^•^–[N_3_]^•^, and (b) the overall reaction **II** + **I** + BrN_3_ → **IV-t** is a Br-atom transfer reaction. Because triplet state intermediate **IV-t** forms from the singlet state reactants, it can form only via the singlet–triplet state seam of crossing. The calculated minimum of the singlet–triplet state seam of crossing, **MECP-1** (see Figure 1), lies only 2.2 kcal/mol higher than that of the reactants. Therefore, it is conceivable to conclude that the formation of **IV-t** is a kinetically very fast process, and spin crossover (from the singlet surface to the triplet surface) takes place as soon as BrN_3_ approaches the metal center.

Subsequent dissociation of the N_3_ radical from **IV-t** to form **V-d** is under entropy control, while slightly exergonic, and leads to the radical (i.e., doublet state) **I**–(phen)Cu^II^(TFA)–Br (**V-d**). In this complex, one unpaired spin is delocalized among the Cu, Br, and O(TFA) centers, and the calculated Cu–Br bond distance is shortened to 2.41 Å (from 2.44 Å in **IV-t**). It is noteworthy that unlike the reported (6,6′-Me_2_bpy)Cu^II^F complex^57^ that quickly dimerized to [(6,6′-Me_2_bpy)Cu^II^F]_2_, the dimerization of (phen)Cu^II^(TFA)Br is unfavorable by 17.9 kcal/mol. In the meantime, the formation of the Br_2_ molecule from (phen)Cu^II^(TFA)Br is highly favorable but, most likely, will proceed via a significant energy barrier, which was not investigated.

To summarize, the data presented above demonstrate that complex (phen)Cu^I^(TFA) preferably reacts with oxidant BrN_3_ and substrate **I** via a Br-atom abstraction pathway that occurs via the singlet–triplet state seam of crossing, is very facile, and leads to **IV-t** and free azide radicals. With the generation of the azide radical, the next step in the mechanism is abstraction of the H atom from the substrate. This process could take place from **IV-t**, which we call the associated mechanism, or from the free azide radical, which we call the dissociated mechanism. Because either intermediate could be important, here we studied both mechanisms for abstraction of the N–H and γ-(C–H) bonds of **I**.

### 3.2 γ-(C–H) Bond Activation by the Free N_3_ Radical

**Figure 2:**
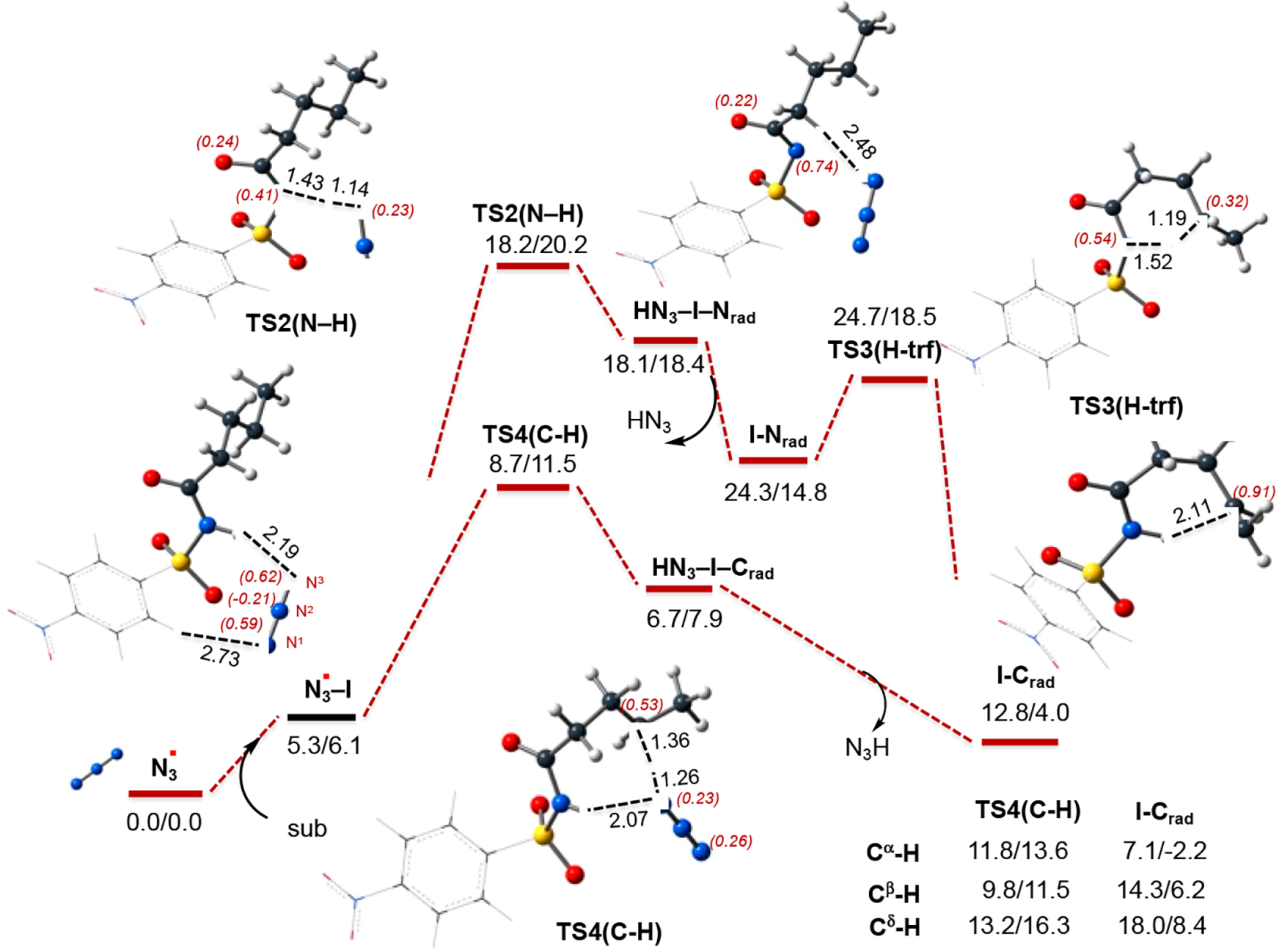
PES of the H atom abstraction from both N–H and γ-(C-H) bonds by free N_3_ radical. Also see the associated energies for α-(C-H), β-(C-H), and δ-(C-H) bonds (in parentheses). The calculated ΔH/ΔG values are in kcal/mol, and bond distances are in Å. The italic numbers in parentheses are the corresponding Mulliken atomic spin density of selected atoms. Color code: black, C; blue, N; white, H; purple, Br; green, F; red, O; and orange, Cu.

The generated free azide N_3_ radical can undergo two obvious transformations. It can react with another azide radical to form three N_2_ molecules.^105^ This process is highly exergonic, occurs very fast, and, probably, is the major (or even only) process involving free N_3_. Regardless, because the reaction of free azide with substrate **I** will shed light on the complexity of the reaction of the Cu-coordinated N_3_ radical with the substrate, below we briefly discuss the abstraction of the H atom from either N–H or C–H bonds in **I** by free azide. Here, we discuss only the abstraction from the γ-(C–H) bond, while we also have calculated the abstraction of H from the α-, β-, and δ-(C–H) bonds (see Figure 2 and the Supporting Information) of **I** by free azide.

In general, the interaction of the substrate with N_3_ is 6.1 kcal/mol endergonic. In the resulting **N**_**3**_**–I** complex, azide is H-bonded to both N–H and C–H bonds; among them, the N_3_–HN (of **I**) distance is shorter than the N_3_–HC (of **I**) distance. Therefore, at first, we discuss the potential energy profile of the abstraction of H from the N–H bond.

As shown in Figure 2, the abstraction of H from N–H occurs with a 20.2 kcal/mol free energy barrier, at transition state **TS2(N–H)**, and leads to the N-centered radical substrate complex HN_3_–**I**–N_rad_. This intermediate, where one unpaired spin is mostly (by 0.74 |e|) located in the N center of substrate **I** with an additional (∼0.22 |e|) spin in carbonyl oxygen, is 18.4 kcal/mol higher in energy than reactants, i.e., N_3_ + **I**. However, it is metastable and quickly dissociates to the HN_3_ molecule and N-centered radical substrate **I–N**_**rad**_. This process is exergonic by 4.0 kcal/mol. The following 1,5-H transfer from the γ-(C–H) bond to the N-radical center of **I–N**_**rad**_ occurs with a 3.7 kcal/mol free energy barrier, at transition state **TS3(H-trf)**, and results in formation of C-centered radical substrate **I–C**_**rad**_. Thus, the path occurring via the N–H bond dehydrogenation and following via the 1,5-H transfer from the γ-(C–H) bond to the N-radical center to form of the required C-centered radical substrate **I–C**_**rad**_ requires overall a 20.2 kcal/mol barrier and is endergonic by 4.0 kcal/mol (see Figure 2). One should emphasize that this path was previously (see Scheme 2) predicted as an initial step of the main mechanistic scenario.

In contrast, the direct abstraction of H from the γ-(C–H) bond of **I** by the azide radical that requires an 11.5 kcal/mol free energy barrier at **TS4(C–H)** that leads to the same C-centered radical substrate **I–C**_**rad**_. It is noteworthy that in **TS4(C–H)** the azide radical remains H-bound with the acidic proton of NH (with a N_3_–HN distance of 2.07 Å), which favorably contributes to the stability of this transition state. Comparison of the calculated energies for both pathways indicates that the N–H site of the substrate is less prone to radical generation. Thus, the abstraction of H from the γ-(C–H) bond, most likely, proceeds via direct C–H dehydrogenation, rather than the previously proposed N–H bond dehydrogenation and then the 1,5-H transfer from the γ-(C–H) bond to the N-radical center. This modified view of C-centered radical substrate **I–C**_**rad**_ formation does not contradict the reported experiments showing no reaction upon replacement of N–H by N–Me. Indeed, as we have shown above, the presence of the N–H bond is critically important because it acts as a directing group, enhancing stabilization of the catalyst–substrate interaction, and recruitment of the substrate to the catalyst.^86^

Thus, here, we identified the direct abstraction of H from the γ-(C–H) bond as a major pathway of the reaction of N_3_ with **I**, rather than the N–H bond as proposed previously. This step of the reaction requires only 11.5 kcal/mol in terms of free energy. Having this conclusion in hand, we also calculated energy barriers and associated transition states for the abstraction of H from the α-, β-, and δ-(C–H) bonds of **I**, which were found to be 13.6, 11.5, and 16.3 kcal/mol, respectively. Comparison of these values with the 11.5 kcal/mol energy barrier reported above for the γ-(C–H) dehydrogenation shows that abstractions of H from the β- and γ-(C–H) bonds of aliphatic amide **I** by the azide radical are kinetically less demanding processes. Because the experimentally observed γ-(C–H) dehydrogenation is thermodynamically slightly less endergonic than the β-(C–H) dehydrogenation (4.0 kcal/mol vs 6.2 kcal/mol), here, we would conclude that the γ selectivity of this reaction with substrate **I** is thermodynamically controlled.

The second stage of the reaction is a bromination of the C-centered radical substrate, **I–C**_**rad**_, by another equivalent of BrN_3_. This reaction, i.e., **I–C**_**rad**_ + BrN_3_ → **I–C–Br** + N_3_, is calculated to be exergonic by 36.3 kcal/mol and proceeds with no energy barrier. The generated second equivalent of the N_3_ radical can initiate the next catalytic cycle of **I** + N_3_ + BrN_3_ → HN_3_ + **I–C**_**rad**_ + BrN_3_ → **I–C–Br** + N_3_.

### 3.3 γ-(C–H) Bond Bromination by the Cu^II^-Coordinated N_3_ Radical

**Figure 3:**
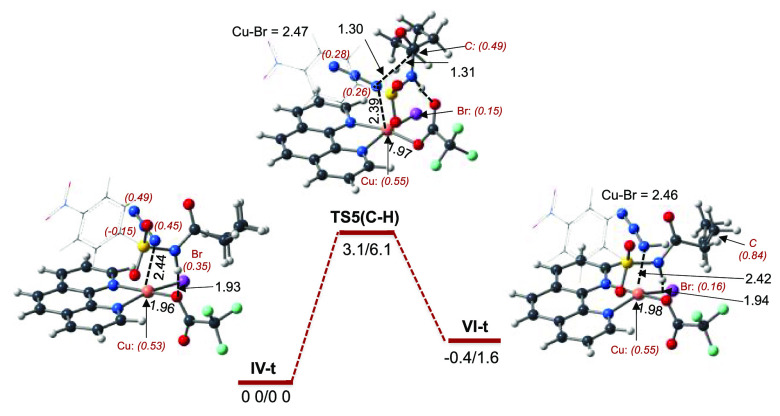
PES of the abstraction of H from the γ-(C–H) bond by the N_3_ radical in **I**–(phen)Cu^II^Br–[N_3_], **IV-t**. The calculated Δ*H*/Δ*G* values are in kilocalories per mole, and bond distances are in angstroms. The italic numbers in parentheses are the corresponding Mulliken atomic spin densities of selected atoms. Color code: black, C; blue, N; white, H; purple, Br; green, F; red, O; and orange, Cu.

To elucidate the impact of the (phen)Cu^I^(TFA) complex (**II**) on the γ-(C–H) bond bromination in **I** presented above, we calculated critical intermediates and transition states of the (a) direct abstraction of H from the γ-(C–H) bond in the **I**–(phen)[Cu^II^Br]^•^–[N_3_]^•^, **IV-t**, and (b) following bromination of the resulting C-centered radical substrate by the Cu-coordinated Br atom. Briefly, as shown in Figure 3, in complex **IV-t**, the abstraction of H from the γ-(C–H) bond occurs with a 6.1 kcal/mol barrier at transition state **TS5(C–H)**. This value of the required barrier is significantly smaller than the value of 11.5 kcal/mol reported above for the reaction of the free azide radical with substrate **I** at transition state **TS4(C–H)** (see Figure 2).

As shown in Figure 3, in the resulting complex **VI-t** the 0.84 |e| α-spin is located in the γ-C-center of the substrate, but the 1.16 |e| spin is delocalized on other atoms with 0.55 and 0.16 |e| spins on the Cu and Br centers, respectively. In the product complex, the formed HN_3_ unit stays coordinated to the Cu center. In the next stage, intermediate **VI-t** dissociates a HN_3_ molecule to form complex **VII-t** and then undergoes bromination of the γ-C center or directly undergoes bromination of the γ-C center. Here we investigated both of these pathways.

**Figure 4:**
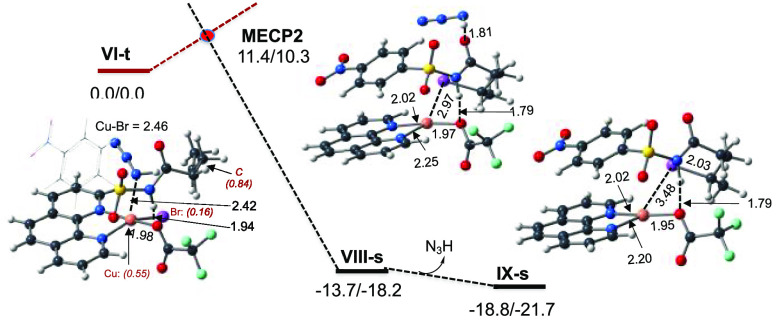
PES of bromination of the C-centered substrate radical by (phen)Cu^I^(TFA)Br···**I**. The calculated Δ*H*/Δ*G* values are in kilocalories per mole, and bond distances are in angstroms. The italic numbers in parentheses are the corresponding Mulliken atomic spin densities of selected atoms. Color code: black, C; blue, N; white, H; purple, Br; green, F; red, O; and orange, Cu.

Briefly, the first pathway requires 13.2 kcal/mol [6.2 kcal/mol free energy for HN_3_ dissociation of HN_3_ and 7.0 kcal/mol energy barrier for the following bromination in **VII-t** (see the Supporting Information)] of free energy. However, direct bromination of the γ-C center in **VI-t** occurs via the singlet–triplet state seam of crossing. The calculated minimum of the singlet–triplet state seam of crossing, **MECP-2** (see Figure 4), lies only 10.3 kcal/mol higher than the reactants and leads to singlet state product **VIII-s**, which rearranges to the energetically most stable complex **IX-s** via dissociation of the HN_3_ molecule. The overall reaction
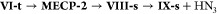
is found to be exergonic by 21.7 kcal/mol.

**Scheme 3:**
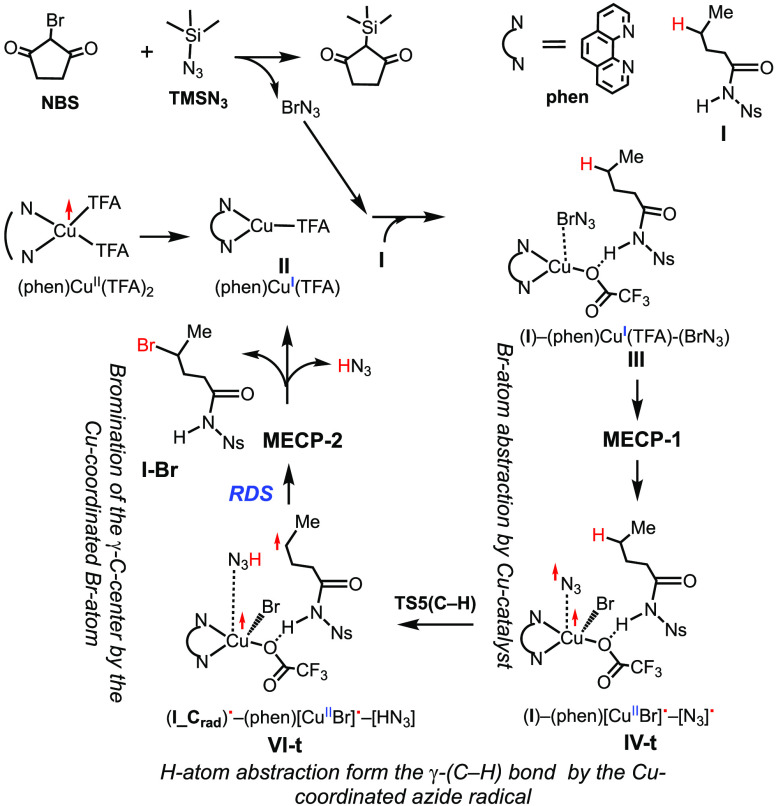
Proposed Modified [from that previously reported (see Scheme 2)] Reaction Mechanism for the Reaction of Cu^I^/Phenanthroline-Catalyzed Selective C–H Bromination Distal to a Directing Functional Group of Substrate *N*-Ns-Pentanamide **I** and by a BrN_3_ Oxidant, Generated *in situ* from NBS and TMSN_3_

On the basis of the discussion presented above, the following modified [from that previously reported (see Scheme 2)] mechanism for the Cu^I^-catalyzed bromination of the γ-[C(sp^3^)–H] bond in *N*-Ns pentanamide **I** in the presence of a phenanthroline (phen) ligand, a 2-bromocyclopentane-1,3-dione (NBS) oxidant, and an azidotrimethylsilane (TMSN_3_) additive was proposed (see Scheme 3):

In the first stage, substrate **I** and oxidant BrN_3_ (generated by the reaction of NBS with TMSN_3_) interact with the Cu^I^ catalyst (**II**) and generate a metastable singlet state complex **III**, where the transfer of the Br atom to the Cu center occurs via an only 2.2 kcal/mol energy barrier at the singlet–triplet state seam of crossing, **MECP-1** (i.e., as soon as BrN_3_ approaches the metal center). In the course of this reaction, the presence of the N–H bond of the substrate is critically important and acts as a directing group to enhance the stability of the catalyst–substrate interaction and recruitment of the substrate to the catalyst.

In the resulting complex **IV-t**, the required C-centered radical substrate formation occurs via direct C–H dehydrogenation by the Cu-coordinated N_3_ radical, rather than the previously proposed N–H bond dehydrogenation and then the 1,5-H transfer from the γ-(C–H) bond to the N-radical center. This step of the reaction, i.e., the **IV-t** → **VI-t** transformation, occurs with a 6.1 kcal/mol free energy barrier and is endergonic by only 1.6 kcal/mol.

The following bromination of the γ-C-radical center by the Cu-coordinated bromine completes the product formation. The transformation of triplet state **VI-t** to singlet state product complex **VIII-s** occurs with a 10.3 kcal/mol energy barrier at the **MECP-2** singlet-to-triplet seam of crossing point and is exergonic by 18.2 kcal/mol. The final stages of the reaction (which are the HN_3_ molecule and the brominated substrate, **I**-Br, dissociation) are kinetically and thermodynamically facile and complete the catalytic cycle.

The rate-limiting step of the entire reaction is the bromination of the γ-C-centered **VI-t** radical complex, while the C–H bond activation by the azide radical is a regioselectivity-controlling step.

Thus, the generated (phen)Cu(TFA) complex **II** plays multiple roles during the reaction. It (a) facilitates generation of the reactive N_3_ radical from BrN_3_ via the Br-atom abstraction scenario, (b) coordinates the N_3_ radical and substrate **I** to initiate the abstraction of H from the γ-(C–H) bond of **I** (coordination of N_3_ to the Cu center reduces the probability of its involvement in the N_2_ formation site reaction), and (c) acts as a bromine source in the following γ-C-radical bromination.

In summary, we found that the reported Cu^II^/phenanthroline-catalyzed C(sp^3^)–H bromination with distal site selectivity starts with the transfer of the Br atom from BrN_3_, which is generated *in situ* from NBS and TMSN_3_ precursors, to the Cu center. This process occurs via a small energy barrier at the singlet–triplet state seam of crossing. In the course of this reaction, the N–H bond of the substrate plays the role of the directing group, and the following C-centered radical substrate formation occurs via the direct C–H bond dehydrogenation by the Cu-coordinated N_3_ radical. This finding is different from the previously proposed N–H bond dehydrogenation and then the 1,5-H transfer from the activated C–H bond to the N-radical center pathway. The C–H bond activation with the azide radical is a regioselectivity-controlling step. The following bromination of the C-centered radical by the Cu-coordinated bromine is the rate-limiting step. Thus, our mechanistic study has identified role of the BrN_3_ molecule and the importance of the azidyl radical that was generated by one-electron oxidation of the LCu^I^(TFA) catalyst. Therefore, it is conceivable to hypothesize that some other Br-containing reagents with an electron affinity similar to that of the BrN_3_ molecule can also be successful brominating reagents and, more critically, safer alternatives to BrN_3_ for remote C(sp^3^)–H bromination of aliphatic amines.

## Supporting Information Available

Structure and relative energies of the calculated (phen)CuII(TFA)_2_ monomer and dimer species (Figure S1), PES of free azide formation upon reaction of (phen)CuII(TFA)_2_ with an oxidant (Figure S2), successive coordination of the substrate and oxidant to (phen)CuII(TFA) (Figure S3), energies of all reported key stationary points (Table S1), and Cartesian coordinates of all reported structures (PDF)
Additional data (XYZ)

## Supplementary Material

om2c00554_si_001.pdf

om2c00554_si_002.xyz
