# Supplementary material for: Mechanism and Selectivity of Copper-Catalyzed Bromination of Distal C(sp3)–H Bonds
Source: Organometallics. 2023 Feb 23;42(18):2467–76. doi: 10.1021/acs.organomet.2c00554 (PMC10526628; doi:10.1021/acs.organomet.2c00554)
Supplement: Supplementary file 1 — om2c00554_si_001.pdf [file om2c00554_si_001.pdf]

SUPPORTING IFORMATION

Mechanism and Selectivity of Copper-Catalyzed Bromination of Distal C(sp^3)-H Bonds

Manjaly J. Ajitha, Brandon E. Haines,* and Djamaladdin G. Musaev*

[1] Cherry L. Emerson Center for Scientific Computation, and Department of Chemistry, Emory University, 1515 Dickey Drive, Atlanta, GA, 30322, United States of America

E-mail: dmusaev@emory.edu
bhaines@westmont.edu

Contents

1. Comment on the used Computational Procedure……………………………………….S2
2. **Figure S1.** The calculated (phen)$Cu^{II}(TFA)_2$ monomer and dimer species. The presented energies are in kcal/mol, and spin densities are in |e| …………………………………..S2
3. **Figure_S2.** The calculated PES of the free azide formation upon reaction of (phen)$Cu^{II}(TFA)_2$ with BrN_3 oxidant. Energies are given in kcal/mol and distances are in Å. ………………………………………………………………………………….S2
4. **Figure S3.** Successive coordination of substrate and BrN_3 oxidant to the (phen)Cu^{II}(TFA) (a) monomer and (b) dimer. Energies are given in kcal/mol ········· S3
5. **Table S1.** Energy parameters (a.u.) of all reported stationary points. ············· S4

Comment on the used Computational Procedure:

All reported structures were fully optimized without any geometry constrains (i.e. all 3N-6 degrees of freedom of each manually generated input structures were optimized) at the (B3LYP-D3)/Gen1 level of theory the Gaussian 09 quantum chemistry package (see main text for more details). Extensive conformational analyses were performed by manual rotation of key bonds and full optimization of all degree of freedoms. We should make it clear that we did not perform any automated conformational analyses nor Boltzmann average the structures.

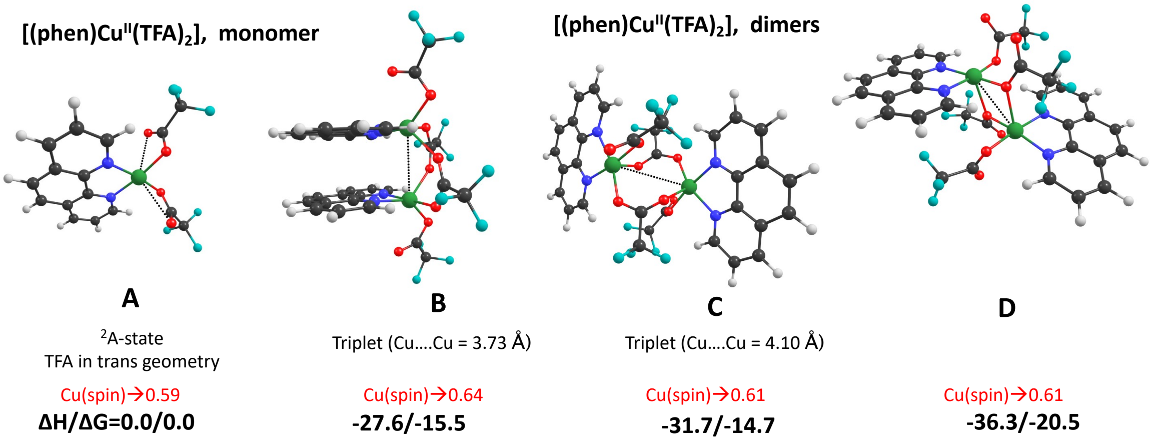

Figure S1. The calculated (phen)Cu^{II}(TFA)$_2$ monomer and dimer species. The presented energies are in kcal/mol, and spin densities are in |e|

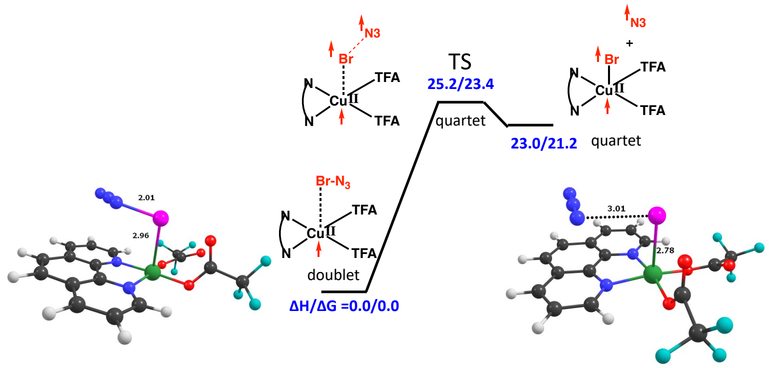

Figure_S2. The calculated PES of the free azide formation upon reaction of (phen)Cu^{II}(TFA)$_2$ with BrN_3 oxidant. Energies are given in kcal/mol and distances are in Å.

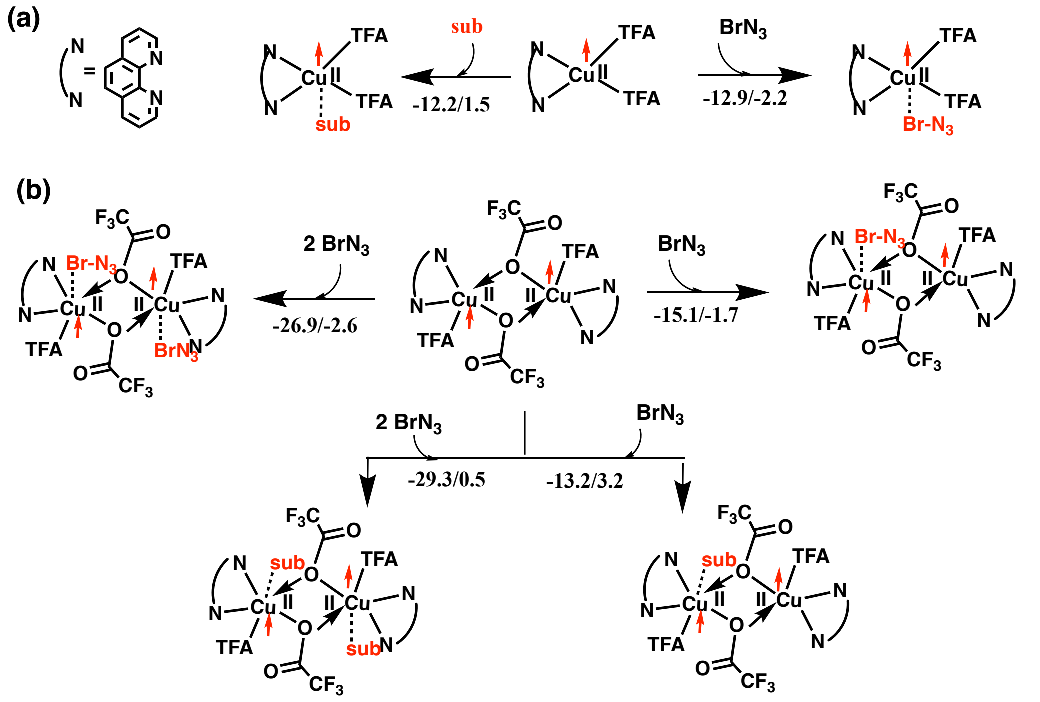

Figure_S3. Successive coordination of substrate and BrN_3 oxidant to the [(phen)Cu^{II}(TFA) (a) monomer and (b) dimer. Energies are given in kcal/mol.

Table S1. Energy parameters (a.u) of all key stationary points. Optimized SCF energies (E_{Gen1}), enthalpy (H_{corr}) and entrolpy (G_{corr})corrections, and single point energies (E_{Gen2}).

	E_{Gen1}	H_{corr}	G_{corr}	E_{Gen2}	H	G	ΔH	ΔG
(phenCu^I(TFA), **II**	-1294.08778	0.22056	0.14977	-1295.63057	-1295.41001	-1295.48081	0.0	0.0
BrN_3	-2735.54995	0.01698	-0.01596	-2738.34697	-2738.32998	-2738.36292		
Sub, **I**	-1311.33728	0.27316	0.20357	-1311.65958	-1311.38643	-1311.45601		
N_3 radical	-164.14044	0.01258	-0.01027	-164.19243	-164.17985	-164.20271		
N_3H	-164.79111	0.02557	-0.00158	-164.84613	-164.82056	-164.84772		
**I_II**	-2605.45276	0.49649	0.38055	-2607.32446	-2606.82797	-2606.94392	-19.8	-4.5
**II_BrN$_3$**	-4029.65902	0.23982	0.15399	-4033.99576	-4033.75594	-4033.84177	-10.0	1.2
**III**	-5341.02277	0.51579	0.3864	-5345.68598	-5345.17019	-5345.29958	-27.5	0.1
**TS1(OA)**	-5340.99594	0.51387	0.38237	-5345.66320	-5345.14932	-5345.28083	-14.4	11.9
**IV-s**	-5341.01953	0.51613	0.38822	-5345.68350	-5345.16737	-5345.29528	-25.7	2.8
**IV-t**	-5341.03834	0.51560	0.38645	-5345.70699	-5345.19138	-5345.32054	-40.8	-13.0
**V-d**	-5176.88843	0.50009	0.37874	-5181.50472	-5181.00463	-5181.12598	-36.4	-18.2
**I-N$_3$**	-1475.48642	0.28816	0.20528	-1475.85761	-1475.56945	-1475.65234	-21.4	-11.4
**TS4(C–H)**	-1475.46574	0.28208	0.20311	-1475.83804	-1475.55596	-1475.63492	-12.9	-0.4
**HN$_3$–I–C$_{rad}$**	-1475.47364	0.28673	0.20526	-1475.84747	-1475.56074	-1475.64221	-15.9	-5.0
**I–C$_{rad}$**	-1310.67012	0.25851	0.18815	-1310.99318	-1310.73467	-1310.80502	-12.5	-11.6
**TS2(N–H)**	-1475.45063	0.28214	0.20187	-1475.82072	-1475.53858	-1475.61885	-2.0	9.6
**HN$_3$–I–N$_{rad}$**	-1475.45597	0.2873	0.20432	-1475.82819	-1475.54089	-1475.62387	-3.5	6.5
**I–N$_{rad}$**	-1310.65252	0.25924	0.18775	-1310.97289	-1310.71365	-1310.78514	0.7	0.9
**TS3(H–trf)**	-1310.64505	0.25447	0.18657	-1310.9659	-1310.71143	-1310.77934	2.1	4.5
**IV-t'**	-5341.03615	0.51544	0.38684	-5345.7055	-5345.19006	-5345.31866	-39.9	-11.9
**TS5(C–H)'**	-5341.02121	0.50896	0.38399	-5345.69101	-5345.18205	-5345.30703	-34.9	-4.6
**VI-t'**	-5341.0295	0.51354	0.38445	-5345.70033	-5345.18679	-5345.31588	-37.9	-10.1
**IV-t**	-5341.03834	0.5156	0.38645	-5345.70699	-5345.19138	-5345.32054	-40.8	-13.0
**TS5(C$_\gamma$–H)**	-5341.02696	0.50899	0.38482	-5345.69556	-5345.18656	-5345.31074	-37.7	-6.9
**VI-t'**	-5341.03291	0.51347	0.38762	-5345.7055	-5345.19204	-5345.31788	-41.2	-11.4
**TS5(C$_\alpha$–H)**	-5341.01538	0.5094	0.38253	-5345.68615	-5345.17675	-5345.30362	-31.6	-2.4
**TS5(C$_\beta$–H)**	-5341.02201	0.50962	0.38404	-5345.69061	-5345.18099	-5345.30657	-34.2	-4.3
**TS5(C$_\delta$–H)**	-5341.0184	0.50902	0.38347	-5345.68766	-5345.17864	-5345.30419	-32.8	-2.8
**VII-t**	-5176.2102	0.48634	0.37214	-5180.83239	-5180.34605	-5180.46025	-25.2	-5.2
**IX-s**	-5176.26748	0.48807	0.36887	-5180.87364	-5180.38558	-5180.50477	-50.0	-33.1
**VIII-s**	-5340.68213	0.51621	0.38925	-5345.73016	-5345.21395	-5345.33789	-54.9	-25.8
